# Physical Activity of the Population of the Most Obese Country in Europe, Hungary

**DOI:** 10.3389/fpubh.2020.00203

**Published:** 2020-06-02

**Authors:** Éva Bácsné Bába, Gergely Ráthonyi, Anetta Müller, Kinga Ráthonyi-Odor, Péter Balogh, Róza Ádány, Zoltán Bács

**Affiliations:** ^1^Institute of Sport Management, University of Debrecen, Debrecen, Hungary; ^2^Institute of Applied Informatics and Logistics, University of Debrecen, Debrecen, Hungary; ^3^Institute of Sectoral Economics and Methodology, University of Debrecen, Debrecen, Hungary; ^4^MTA-DE Public Health Research Group, Public Health Research Institute, University of Debrecen, Debrecen, Hungary; ^5^Department of Accounting, Institute of Accounting and Finance, University of Debrecen, Debrecen, Hungary

**Keywords:** international physical activity questionnaire, physical activity, cluster analysis, Hungary, obesity, metabolic equivalent

## Abstract

**Introduction:** Physical activity is inversely proportional to mortality, so it has an important role in disease prevention. The aim of our study was to characterize the physical activity of Hungarians, the most obese population in Europe.

**Materials and methods:** In a cross-sectional study the physical activity of the Hungarian population was characterized in a sample (*n* = 1,295) which was representative of the sex, age and geographical location of the adult population aged 18 years and above by using the long form of the International Physical Activity Questionnaires (IPAQ) as an instrument. Based on the metabolic equivalent (MET) rates three categories of physical activity (low, moderate, and high) were defined. Two-step cluster analysis was used to explore physical activity characteristics of participants using sex, age, settlement type and BMI categories as categorical variables, and MET values related to the Work, Transportation, Domestic and Garden, and Leisure Time domains of physical activity as continuous variables.

**Results:** The study showed that 63.39% of the adult Hungarian population took part in high, and 24.78% in moderate activity, and only 11.73% of the sample belonged to the category of low physical activity. By cluster analysis six clusters of people with typical lifestyles could be identified in the Hungarian adult population. In all the six groups participants achieved moderate or high activity levels through work and housework. Physical activity in relation to transportation is very low, similarly to leisure-time sporting activities. In the case of elder people, severe overweight/obesity problems can be detected in married city-dwellers.

**Discussion:** Although Hungary has the highest obesity rate in Europe our research has proved that Hungarians lead physically active lives. The dominant forms of their physical activity are linked to work and housework. Our findings draw attention to the need to examine other risk factors in addition to physical inactivity. Our findings also suggest that the type of physical activity should be more severely considered when defining factors protective against obesity.

## Introduction

Obesity is considered to be the pandemic of the 21st century (1), showing a growing prevalence in all ages and both sexes irrespective of geographical locality, ethnicity or socioeconomic status (2). As the World Health Organization reported more than 1.9 billion adults aged 18 years and older were overweight in 2016 (3). Statistical figures for 2016 published by the OECD show that Hungary has the highest obesity rate in Europe, and only the populations of the United States, Mexico and New-Zealand are more obese than Hungarians (4). Most recently the Hungarian Central Statistical Office reported that 55.5% of Hungarians aged 15 years and older are obese or overweight (5). The premature mortality caused by non-communicable diseases (NCDs) strongly related to obesity is very high in Hungary, and average life expectancy at birth lags almost 5 years below the EU average (6).

Although obesity is a complex multifactorial phenotype, it is generally accepted that it occurs mainly because of an imbalance between energy intake from the diet and energy expenditure through physical activity, i.e., the obesity epidemic is most strongly related to unhealthy diet and physical inactivity (7).

Around the world, annually nearly 5 million people die of physical inactivity (8). An abundant number of studies confirm that physical activity is inversely proportional to mortality and has a number of health benefits (9–11), notably in relation to cardiovascular (12–14), malignant (15, 16), metabolic (17–20), locomotor (21–25), and mental (26–28) disorders.

Measuring physical activity is a real challenge for public health studies (29), but it is generally accepted that the International Physical Activity Questionnaire (IPAQ)—designed to be easily adapted for many languages and countries—can be used to obtain internationally comparable data on health–related physical activity by assessing practically all domains of physical activity (leisure or recreational activities, activities of people in their jobs, activities through the type of transportation they use, and in performing household chores) and it can be applied effectively in cross-sectional studies at population level (30, 31).

The aim of our study was to characterize the physical activity of the Hungarian population with a survey designed to cover all areas of physical activities. With respect to the Hungarian population, physical activity had never before been assessed with the use of the most broadly accepted assessment tool, the International Physical Activity Questionnaires (IPAQ) long form on a representative sample, and therefore our results—in addition to providing a detailed characterization of the physical activity of the population with the highest prevalence of obesity in Europe—also have the potential to contribute to the planning of targeted preventive interventions.

## Materials and Methods

### Study Sample

A total of 1,343 participants aged 18 years and older were recruited with the help of a market research company, Synapsis, Debrecen, Hungary. The company ensured the representativeness of the sample according to four characteristics: sex, age, type of settlement and region. The online survey was designed to protect respondent anonymity. The study was approved by the Regional Ethics Board (DE RKEB/IKEB: 4842-2017) at the Clinical Center of the University of Debrecen. All participants provided informed consent in compliance with the principles of the Declaration of Helsinki and the General Data Protection Regulation (GDPR).

### Instrument

The International Physical Activity Questionnaire (IPAQ) long form was applied. This questionnaire is designed to assess the time spent walking, doing moderate-intensity and vigorous-intensity activity according to different domains: [1] work, [2] transport, [3] domestic and garden, [4] leisure, and [5] time spent sitting in the last 7 days. The personal and socio-demographic characteristics of the participants (sex, age, settlement type, region, marital status, education and occupation, household income, height and body weight) were also recorded in the self-reported online questionnaire.

### Data Collection

Data collection was conducted by the market research company (Szinapszis Market Research& Consulting Ltd, Debrecen, Hungary) via self-reported questionnaires from 1st November 2017 to 31st January 2018. Computer-assisted web interviewing (CAWI) and computer-assisted telephone interviewing (CATI) methods were used by the company, which was paused from 20th December to 8th January in order to exclude the Christmas holidays to minimize the effects of seasonal bias on data related to physical activity. All IPAQ data was processed by using the standardized IPAQ Scoring Protocol. During the data cleaning, outlier and non-real values were excluded. Subsequently, there remained records from 1295 participants in the final database, which were used for further statistical analysis. Data were stratified by sex- and age-groups, as well as by the type of settlements where the participants lived. Weight and height values were used to calculate the body mass index (BMI). BMI is commonly used to classify underweight, overweight and obesity in adults. It is defined as the weight in kilograms divided by the square of the height in meters (kg/m^2^). The classification of adults according to BMI is: Underweight <18.50; Normal range 18.50–24.99; Overweight: 25.00–29.99; Obese > 30 kg/m^2^ (32).

In the analysis of physical activity responses were converted to Metabolic Equivalent Task minutes per week (MET-min/week) according to the IPAQ scoring protocol (30): i.e., the total minutes over the last 7 days spent on different types of physical activity to create MET scores for each activity category. MET scores across the several physical activity sub-components were analyzed and they were summed to indicate overall physical activity. According to the metabolic equivalent (MET) rates three physical activity categories (low, moderate, high) were defined. The items in the long IPAQ form were structured to provide separate domain specific scores for walking, moderate-intensity and vigorous-intensity activity within each of the work, transportation, domestic chores and gardening (yard), and leisure-time domains. The total scores for the long form are the sum of the duration (in minutes) and frequency (days) for all the types of activities in all domains. Domain specific scores or activity specific sub scores could also be calculated by summing the scores for walking, moderate-intensity and vigorous-intensity activities within the specific domain, whereas activity-specific scores are the sum of the scores for the specific type of activity across domains.

Data collected with the IPAQ long form was reported as a continuous measure and reported as median MET-minutes. Median values and interquartile ranges were computed for walking (W), moderate-intensity activities (M), and vigorous-intensity activities (V). Total scores were also calculated for walking (W), moderate-intensity activities (M), and vigorous-intensity activities (V); for each domain (work, transport, domestic and garden, and leisure) and for an overall grand total (33).

Data collected with IPAQ was reported as categorical variable as well. These categories are:

1. Low physical activity,
2. Moderate physical activity,
3. High physical activity of population.

As the IPAQ Guideline defines the “low” category as the lowest level of physical activity; those individuals who not meet criteria for categories “moderate” or “high” are considered to have a “low” physical activity level.

The pattern of activity to be classified as ‘moderate’ is either of the following criteria:

-3 or more days of vigorous-intensity activity of at least 20 minutes per day

OR

-5 or more days of moderate-intensity activity and/or walking of at least 30 minutes per day

OR

-5 or more days of any combination of walking, moderate-intensity or vigorous intensity activities achieving a minimum total physical activity of at least 600 MET-minutes/week.

Individuals meeting at least one of the above criteria would be defined as accumulating a minimum level of activity and therefore be classified as “moderate.”

The “high” category is a separate category to describe higher levels of participation.

The two criteria for classification as “high” are:

– vigorous-intensity activity on at least 3 days achieving a minimum Total physical activity of at least 1500 MET-minutes/week

OR

-7 or more days of any combination of walking, moderate-intensity or vigorous-intensity activities achieving a minimum total physical activity of at least 3000 MET-minutes/week (33).

As a conceptually new analytical approach the MET distribution of physical activity categories (as low, moderate and high) was constructed by calculating and summing the MET values of the different physical activity domains and the domain with the highest contribution was identified. In the further analysis subgroups were defined on the basis of the dominant domain (work, transportation, domestic and garden, and leisure time).

### Statistical Analysis

Statistical analysis was performed using SPSS v21.0. In all statistical tests, statistical significance was defined as *p* < 0.05. The frequency of physical activities was calculated by type in the dimension of the different domains. A Pearson's Chi-square test was used to investigate the association between physical activity and individual as well as socio-demographic characteristics. The following three paragraphs are inserted into the Methods section:

In order to assess the number of respondents falling into each cluster, the adjusted residuals (ARs) were defined. When the AR is more than 2.0 it is supposed that a significantly higher number of respondents are in a cluster specified than would be expected if the null hypothesis were true. When the adjusted residual falls below – 2.0 it is supposed that a significantly lower number of respondents are in certain cluster than would be expected if the null hypothesis were true.

The two-step cluster analysis procedure was carried out to explore physical activity characteristics of participants using sex, age, settlement type and BMI categories as categorical variables, and Work, Transport, Domestic and Garden, and Leisure MET values as continuous variables.

The two-step cluster analysis is used for the analysis of databases consisting of large numbers of elements, when hierarchic or K-means clustering is less effective. This analytic method can be applied both to categorical and continuous variables. The two-step cluster analysis uses the two-step approach where the first step in the algorithm results in pre-clusters in which the respondents are sequentially grouped by constructing a cluster feature tree. The second step takes the pre-clusters from the previous step, and arranges them into the final clusters using the hierarchical clustering method where the pre-clusters are recursively merged. To determine the cluster numbers, the Bayesian information criterion (BIC value) and the ratio of distance measures was used (34). Two distance measures were employed namely, log-likelihood (for categorical variables) and Euclidean (for continuous variables). To test the stability of the clusters the cluster analysis procedures were repeated in internal random samples consisting of 50% of the total study sample by using Blashfield and Macintyre's split sample method. Cohen's kappa coefficient was calculated to measure the agreement between their equivalent clusters (35). The internal consistency of the clusters was examined for physical activity parameters.

A Pearson's Chi-square test was used to investigate the association between clusters (36), in the dimension of physical activity and personal and socio-demographic characteristics.

## Results

### Characteristics of the Sample

In the sample assessed after data cleaning (*N* = 1,295), 47.7% (618 persons) were male and 52.3% (677 persons) female subjects. Regarding age distribution, in the sample 28.3% (251 persons) belonged to the 18–29 years age group, 39.08% (506 persons) to the 30–49 years age group, 16.06% (208 persons) to the 50–59 years age group and 25.48% (330 persons) were over the age of 60. Concerning the settlement type 17.3% (224 persons) lived in the capital, Budapest, 25.2% (326 persons) in county town, 37.4% (484 persons) in other cities and towns, and 20.1% (261 persons) in villages. The regional distribution of the participants involved: 30% (389 persons) came from Budapest and its surrounding region, 44.3% (574 persons) from East Hungary and 25.6% (332 persons) from West Hungary (Table 1).

**Table 1:** Distribution of the sample by sex, age, education, settlement type, regions, marital status, household income, daily working hours, BMI and the average age of the sample with standard deviation.

		**Total (*n* = 1,295)**	**%**
Sex	Male	618	47.7
	Female	677	52.3
Age group (years)	18–29	251	19.4
	30–39	239	18.5
	40–49	267	20.6
	50–59	208	16.1
	60+	330	25.4
	Average age (SD)	45.9 (15.2)	
Education	Maximum primary school,	156	12.1
	Secondary, high school education	601	46.4
	College/University, degree	538	41.5
Settlement type	Capital city	224	17.3
	County town	327	25.3
	City	484	37.4
	Rural area	260	20.1
Household income^*^	<150 000 HUF (<429 EUR)	266	20.5
	150 001–300 000 HUF (429–857 EUR)	472	36.5
	300 001–500 000 HUF (857–1429 EUR)	279	21.5
	More than 500 000 HUF (>1,429 EUR)	109	8.4
	No answer	169	13.1
Working hours	8 h	737	56.9
	Less than 8 h (part-time employment)	355	27.4
	More than 8 h (frequent overtime)	190	14.7
	No answer	12	0.9
Marital status	Unmarried / Single	327	25.3
	Married and living together (including registered partnership)	724	55.9
	Married but live separately	32	2.5
	Widowed	69	5.3
	Divorced (including legally terminated partnership)	143	11.0
	Budapest—Pest county	389	30.0
Region	East-Hungary	575	44.4
	West-Hungary	331	25.6
	Underweight (<18.50)	92	7.1
BMI (kg/m^2^)	Normal weight (18.50–24.99)	461	35.6
	Overweight (25.00–29.99)	436	33.7
	Obesity (>30.00)	306	23.6

* *1 EUR, 350 HUF*.

In the sample assessed, it was found that 63.39% of the adult Hungarian population engaged in vigorous and 24.78% in moderate activity, and only 11.73% of the sample belonged to the inactive (low activity) category.

Table 2 demonstrates differences in physical activity with respect to sex, age, education, settlement type and region. Male respondents with high activity levels are significantly more numerous (67.3%) than women (60%). Regarding levels of physical activity of respondents, our findings show significant differences in connection with education, settlement type, and region.

**Table 2:** The association between physical activity and personal/socio-demographic characteristics of the sample.

		**Low (*n* = 152)**	**Moderate (*n*= 322)**	**High (*n* = 821)**	**P for trends**
Sex	Male	67	136	415	0,021^**^
	Female	85	186	406	
Age group (years)	18–29	31	71	149	0.609
	30–39	33	63	143	
	40–49	29	59	179	
	50–59	24	53	131	
	60+	35	76	219	
Education	Maximum primary school,	27	21	108	0.000^***^
	Secondary, high school education	58	131	412	
	College/University, degree	67	170	301	
Settlement type	Capital city	24	77	123	0.002^**^
	County town	48	84	195	
	City	53	110	321	
	Rural area	27	51	182	
Region	Budapest—Pest county	37	121	231	0.002^**^
	East-Hungary	64	125	386	
	West-Hungary	51	76	204	

*** *p < 0.001*,

** *p < 0.05*.

In the light of socio-demographic data, a significant difference in the level of leisure-time activity can only be found with respect to sex; a significantly higher proportion of men (23%) than women (17.5%) belong to the high-activity category.

In the distribution of MET values and the physical activity categories by domain (Table 3) significant differences were observed (*p* <0.001). In the group of those reporting vigorous activity, the given activity levels are achieved through work by 48% of the respondents (390 persons), through housework by 25% (208 persons), and by only 23% (189 persons) through leisure sports. Those respondents doing moderate activity mostly reached the stated level of activity though housework (40%−129 persons) and work (25%−79 persons). On the basis of these findings, it can be stated that physical activity in the Hungarian population is mainly the result of work and housework.

**Table 3:** The relationship between the physical activity domains and the physical activity categories defined on basis of MET values.

**Domains**	**Categories of physical activity**	**Low (*n* = 152)**	**Moderate (*n* = 322)**	**High (*n* = 821)**	**Total (*n* = 1,295)**
Work		42	80	390	512
Transport		14	44	34	92
Domestic and garden		62	129	208	399
Leisure		34	69	189	292
Total		152	322	821	1,295

**Figure 1:**
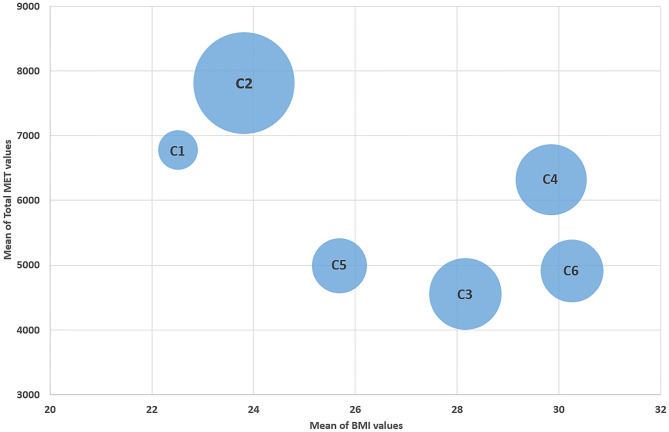
Presentation of the clusters as a function of “BMI” and “Total MET” values. C1, Cluster 1 “Yet to leave the nesters”; C2, Cluster 2 “Working mothers”; C3, Cluster 3 “Aging but not slowing down”; C4, Cluster 4 “Physically active city-dwellers”; C5, Cluster 5 “Trendy Women”; C6, Cluster 6 “Super Grandma”.

The number of clusters was defined on the basis of the best combination of low Bayesian Information Criterion (BIC) and high ratio of BIC changes as well as meaningful conceptual considerations. Change in BIC against number of clusters is shown in Table 4 and the BIC continues to decline considerably until the six-cluster solution (Table 4). The quality of clustering solution was quantified on the total data set by defining silhouette coefficients indicating cohesion and separation (silhouette values ranges from −1 to +1; a relatively high value indicates that the person is well-matched to its own cluster and poorly matched to neighboring clusters) (Figure 1).

**Table 4:** Comparison of models by bayesian information criterion and ratio of BIC changes and ratio of distance measures.

**Number of clusters**	**BIC**	BIC change^*^	Ratio of BIC changes^**^	Ratio of distance measures^***^
1	15740.053			
2	14790.698	−949.354	1.000	1.058
3	13900.577	−890.121	0.938	1.211
4	13189.094	−711.483	0.749	1.379
5	12710.261	−478.833	0.504	1.010
6	**12237.324**	**−472.937**	**0.498**	**1.265**
7	11892.002	−345.322	0.364	1.281
8	11652.159	−239.843	0.253	1.027
9	11422.224	−229.934	0.242	1.205
10	11254.470	−167.755	0.177	1.020
11	11092.678	−161.792	0.170	1.097
12	10957.293	−135.385	0.143	1.097
13	10845.839	−111.454	0.117	1.070
14	10750.484	−95.355	0.100	1.058
15	10667.823	−82.661	0.087	1.026

*BIC, Bayesian Information Criterion*.

* *The changes are from the previous number of clusters in the table*.

** *The ratios of changes are relative to the change for the two cluster solution*.

*** *The ratios of distance (log-likelihood) measures are based on the current number of clusters against the previous number of clusters. Bold values represents the six cluster solution – the most appropriate model*.

The silhouette measure of cohesion and separation was above 0.5, indicating good cluster quality. Conducting a replication analysis using Blashfield and Macintyre's split sample method produced clusters that were fairly similar to the main results. Cohen's kappa coefficient was 0.43 (P <0.001), suggesting “moderate” agreement, i.e., the agreement is far from 80% interpreted as the minimum acceptable interrater agreement.

Cluster analysis was used to divide up the sample on the basis of sex, age, place of residence, region, BMI index, family status, activity level, physical activity categories and leisure-time activity (Table 5). The following clusters could be determined:

1. Men aged between 18 and 29 years, living exclusively in cities, who are the most likely to have optimum body weights, and the most of them are singles. The dominant proportion of dose belonging to this cluster are fairly active, and this activity is mostly present in their work and leisure-time. The members of this group show a considerably higher level of leisure-time sporting activity (49% of them do sports) than the EU average from the 2017 Eurobarometer survey (37) or than the Hungarian average. On the other hand, housework is not typical of this group. This cluster has been named “Yet to leave the nesters.”
2. Most women of 30–39 years or younger, who predominantly live in small towns and villages; the majority have a normal body mass index, are married or have partners, and can be described as engaging in a high level of physical activity that is mostly achieved through work and housework. 64% of them have no time to do sports. This cluster has been named “Working mothers.”
3. Mostly men over the age of 60 who live in county towns, who belong to the overweight or obese category, and the majority of whom are married or have partners. The greater proportion of them engage in a high level of physical activity, which is primarily achieved through their work, and 40% of them also do sports. This cluster has been named “Aging but not slowing down.”
4. Men who are over the age of 50 or 60 years, live in cities and towns, belong to the overweight or obese category, are rather married or have partners, and engage in high or moderate physical activity, which is achieved through work or housework. 67% of them do not do any leisure sports. This cluster has been named as “Physically active city-dwellers.”
5. Most of the women being married or have partners who live in cities or the capital city are over the age of 50 years and have normal body weights. They show predominantly high physical activity achieved with housework or work, whereas their activity in connection with transport is low. This cluster has been named as “Trendy women.” (“Taxi mums” is a Hungarian term for mothers who take their children to special classes and training sessions by car).
6. Women who are predominantly city-dwellers, most of whom are over the age of 60, but all of whom are over 40, and nearly all of whom (99%) are overweight or obese. The majority are married or have partners, most of them engage in a high level of physical activity, which is primarily achieved through housework. This cluster has been named “Super Grandma.”

**Table 5:** Difference in the six clusters on the basis of sex, age, place of residence, region, BMI index, family status, activity level, physical activity categories and leisure-time activity.

		**Yet to leave the nesters *n* = 68**	**Working mothers *n* = 445**	**Aging but not slowing down *n* = 224**	**Physically active city-dwellers *n* = 217**	**Trendy women *n* = 132**	**Super Grandma *n* = 169**	**Chi-square**
Sex	Male	68	160	135	217	24	0	551.026^***^
	Female	0	285	89	0	108	169	
Age group	18–29	60	145	17	25	0	0	
	30–39	2	161	35	34	0	0	874.691^***^
	40–49	0	103	66	40	0	49	
	50–59	6	16	29	44	80	26	
	60+	0	20	77	74	52	94	
Settlement type	Capital city	16	77	0	42	41	38	939.673^***^
	County town	25	50	224	0	19	0	
	City	27	162	0	137	51	91	
	Rural area	0	156	0	38	19	40	
BMI categories	Underweight (<18.50)	7	67	2	0	15	0	656.518^***^
	Normal weight (18.50–24.99)	61	256	54	0	81	1	
	Overweight (25.00–29.99)	0	79	106	131	0	103	
	Obesity (>30.00)	0	43	63	86	36	65	
Marital status	Unmarried/Single	48	171	38	42	11	7	327.119^***^
	Married and living together (including registered partnership)	8	194	128	134	70	101	
	Married but live separately	1	9	10	3	6	3	
	Widow	1	5	11	8	16	26	
	Divorced (including legally terminated partnership)	1	25	33	23	26	33	
	Relationship	10	40	4	8	1	0	
Moving categories	Low	7	52	28	19	11	15	6,866
	Moderate	13	106	58	54	35	50	
	High	48	287	137	144	85	105	
Type of the MET	Work	28	183	89	101	45	46	71.585^***^
	Transport	8	33	14	17	9	6	
	Domestic and garden	8	122	54	50	51	88	
	Leisure	23	100	62	46	25	28	
Leisure categories	No sports activity	35	284	135	146	94	107	27.772^***^
	Moderate sports activity	9	64	44	25	14	38	
	Vigorous sports activity	24	97	45	46	23	24	
Household income	<150 000 HUF	18	112	40	29	26	35	38.254^***^
	150 001–300 000 HUF	27	150	77	86	49	73	
	300 001–500 000 HUF	13	83	51	60	24	38	
	More than 500 000 HUF	7	43	30	17	8	3	

*** *p < 0.05*.

These clusters represent six active groups with typical lifestyles in the Hungarian adult population. According to our results, in all the six groups they achieve moderate or high activity levels through work and housework. Activity in relation to transport is minimal, while leisure-time sporting activities are at a low level. In the case of older, married city-dwellersmany of them are overweight.

## Discussion

In the Hungarian sample assessed in the study, it was found that 63.39% of the adult Hungarian population engaged in vigorous and 24.78% in moderate activity, and only 11.73% of the sample belonged to the inactive category. The results of our study were similar to the findings in a Slovenian survey (38), where 12.2% of the respondents were found to be physically almost inactive. According to Guthold et al. (39) globally more than one-fourth of adults can be described as insufficiently physically active in comparison the Hungarian population seems to show more favorable results.

In our research there was a larger proportion of people who have a high level of activity living in villages (70%) than in the capital city (54.9%) and county towns (59.6%). Village lifestyles involve more housework, gardening, and participation in pedestrian or cycling transport in day-to-day life than in urban environments. This corresponds to the study findings of Dumith et al. (40), where it was claimed in relation to the comparison of different countries that the population of more urbanized countries showed lower levels of physical activity. A Croatian study (41) pointed out that the population of the capital city (Zagreb) was the most inactive in comparison to the Croatian national average.

Significant regional differences were also found in the levels of physical activity (*p* < 0.05). In Budapest and its surrounding region, as well as in the West Hungarian Region the proportion of those with high levels of physical activity (59.4 and 61.6%, respectively) was lower than in the Eastern region (67.1%) of the country. As Hungarians achieve these activity levels through work, followed by housework, the results obtained can be partly explained by the fact that the Eastern region has less developed economy with larger areas involved in agricultural cultivation and lower degree of urbanization than the western part of the country, and therefore the proportions of physical work and housework also tend to be greater.

In terms of education, our study found very strong, significant difference among the physical activity categories. Those with higher education qualifications made up a smaller proportion (56%) in the high-intensity range, whereas people with lower levels of schooling (68.6% of those with secondary and 69.2% of people with primary education) tend to be more numerous in the high-activity group. This result can be explained by the fact that a larger proportion of people with higher levels of schooling are involved in intellectual, white-collar jobs requiring sedentary work, and consequently lower workplace activity, while more physical work can be observed among people with lower levels of schooling.

In terms of family status, personal income and the number of working hours spent at the workplace, there were no significant differences found among the physical activity categories of the sample. In their study Hull et al. (42) also observed no differences in the physical activity of young married adults and young adults with children on the one hand, and unmarried young people on the other hand.

The results of our cluster analysis indicate that in the context of a work (in the first 4 clusters) and in domestic and garden activities (in the last 2 clusters) is present in fairly high proportion. In research conducted on a Turkish sample (43), there were no significant differences in physical activity between women and men, but while women tend to show more physical activity in housework, for men work at their workplaces dominates. Malaysian research also confirmed higher activity for men (44), but while male respondents spent more time on work and leisure-time/recreational activities, female respondents proved to be more active in housework/household management. In our study, both men and women show high levels of activity in work (40% in all the clusters), but the dominance of women in housework is confirmed by the last two clusters.

With respect to leisure activity, our results correspond to the Eurobarometer survey of 2017 (37), stating that 56% of the Hungarian adult population never does any sport. According to the Eurobarometer survey of 2017, 40% of Europeans (EU 28) i do sports at least once a week. When compared to the EU average (46%), the 56% of the Hungarian population who never do any sport is fairly large, and shows a 9% increase in comparison to the result obtained in the earlier survey carried out in 2013 (37). Even when a comparison is made with the V4 countries, significantly worse values (*p* < 0.001) have been experienced with respect to physical activity in Slovakia and especially Hungary, as data for their Czech and Polish counterparts are more favorable (45).

Lear et al. (46) conducted a study covering 17 countries of different income levels in various continents, with the involvement of nearly 170,000 respondents from varied social and economic backgrounds, and concluded that the benefits of physical activity were independent of the types of the physical activities, i.e., whether the activities were classified as leisure-time or other activities. However, health recommendations and many studies primarily associate the health benefits arising from physical activity and lower levels of mortality with outdoor, recreational sporting activities (47–52).

Our results indicate that in the context of work and domestic and garden activities, physical activity is present in fairly high proportions. Concerning the fact that the mortality caused by physical inactivity and consequently the prevalence of obesity-related non-communicable diseases are very high in the Hungarian population (53), it is reasonable to suggest the reconsideration of conflicting opinions on the protective role of different types of physical activity.

On the basis of our results, we tend to accept the opinion that the benefits of physical activity are not independent of the types of the physical activity, i.e., whether it is classified as leisure-time or other activity (47–52).

However, the present study has some limitations. These derive from the well-known limitations of cross-sectional studies, in our study the season in which the data was collected may be an issue, and therefore it is possible that cluster membership may be different in another season.

The questionnaire was based on self-reported measures of physical activity which may not accurately reflect physical activity, furthermore, it could result in participants' answers being influenced by social desirability. In contrast with objective measurement of physical activity (e.g., a pedometer), all items of the IPAQ rely on the memory of the participants which can increase the risk of an under or overestimation of physical activities. In the sample the more highly educated respondents were overrepresented because they were rather reachable than lower educated people by telephone and the Internet.

## Conclusion

It has been proved that Hungarians lead active lives, but the dominant forms of their physical activity are work and housework. As recent studies point out that the health benefits of activity emerge independently from the type of activities engaged in, irrespective of whether they are associated with leisure-time or non-leisure activities, the 88.27% proportion of the Hungarian population involved in high or moderate activity can be regarded as an above average result. In the context of prevention strategies, our results underline the need to improve and develop options for physical activities in leisure-time sports and in connection with transport. The overweight problems that are present in parallel to the active lifestyles of the population draw attention to the need to examine other risk factors (especially dietary habits) in addition to physical activity.

## Data Availability Statement

The datasets generated for this study are available on request to the corresponding author.

## Ethics Statement

The studies involving human participants were reviewed and approved by Regional and Institutional Ethics Committee at the Clinical Center of the University of Debrecen. Ethical approval number: DE RKEB/IKEB-4843-2017. The patients/participants provided their written informed consent to participate in this study.

## Author Contributions

ÉB, RÁ, ZB, and AM contributed to the design and implementation of the research. PB, GR, ÉB, and RÁ managed and analyzed the data. ÉB, RÁ, KR-O, and AM contributed to the interpretation of the results. ÉB wrote the manuscript. RÁ, ÉB, and GR edited the manuscript. All authors provided critical feedback and helped shape the manuscript.

## Conflict of Interest

The authors declare that the research was conducted in the absence of any commercial or financial relationships that could be construed as a potential conflict of interest.
